# Anxiety in the Pediatric Cystic Fibrosis Population: Evaluation of a Younger Cohort

**DOI:** 10.1002/ppul.71181

**Published:** 2025-06-26

**Authors:** Jemilla Strode Smith, Josie van Dorst, Michael J. Coffey, Chee Y. Ooi

**Affiliations:** ^1^ School of Clinical Medicine, Discipline of Paediatrics & Child Health, UNSW Medicine & Health University of New South Wales (UNSW) Sydney Australia; ^2^ Department of Gastroenterology Sydney Children's Hospital Randwick Australia

To the Editor,

Mental health disorders have been significantly associated with decreased treatment adherence in people with cystic fibrosis (PwCF), increasing healthcare costs, number of hospitalisations and length of stay [1]. The International Depression Epidemiological Study (TIDES) (2014) reviewed 6088 PwCF aged over 12 years old from cystic fibrosis (CF) centres in Europe and the United States using the Hospital Anxiety and Depression Scale [1]. TIDES identified a global prevalence of anxiety of 22% and 32% in adolescents and adults with CF, respectively, surpassing estimated rates of anxiety in the general population by 2‐3 times [1].

Developed in response to TIDES, international consensus guidelines recommend annual screening of anxiety and depression in PwCF from age twelve, using Patient Health Questionnaire‐9 and General Anxiety and Depression‐7 scale scores [2]. Whilst TIDES found that older age was associated with higher rates of anxiety and depression, the extrapolation of these findings to children under the age of twelve is limited by the TIDES exclusion of this demographic, and the scarce number of studies into anxiety prevalence in this age group. This single‐centre study aimed to identify the prevalence of anxiety in a young cohort of cystic fibrosis children compared to non‐CF controls.

This study is part of the Evaluating the Alimentary and Respiratory Tracts in Health and Disease programme (EARTH); a prospective, longitudinal, observational study of children aged 0–18 years diagnosed with CF, recruited from the CF clinic at Sydney Children's Hospital Randwick, Australia (Ethics number: HREC/18/SCHN/26) [3]. CF participants were recruited from the CF outpatient clinic between 2018 and 2022. Non‐CF controls were recruited from outpatient clinics (primarily orthopaedics and ophthalmology for the treatment of acute injuries), advertisements and word‐of‐mouth. Controls were recruited on the basis of being free from chronic illnesses, including previous mental health conditions. Participants were excluded for inability to comply with study requirements and non‐English speaking guardians [3]. Parental consent was acquired from all participants.

Spence Children's Anxiety Scale questionnaires were electronically distributed to CF and non‐CF control participants. Validated for children aged 0–18, this scale has high internal consistency, convergent and divergent validity [4]. Preschool Anxiety scales and Spence Children's Anxiety Scales (Parent Report) were completed by parents for children aged 0–4 years and 5–8 years, respectively. Spence Children's Anxiety Scales were completed for children greater than 8 years old, where parent's discretion dictated whether child‐reported or parent‐reported surveys were undertaken. Of participants aged over 8 years, parent‐reported surveys were completed by 56% of the CF group and 7% of the non‐CF group.

The Preschool Anxiety Scale questionnaire consists of 28 questions scored from 0 to 4 (representing ‘Not true at all’ to ‘Very often true’) to yield a maximum total score of 112 [5]. Child and Parent‐reported Children Anxiety Scale questionnaires consist of 38 scored questions reported on the basis of symptoms being experienced ‘Never’ (3) to ‘Always’ (3), for a maximum score of 114 [4]. Questions assess both total anxiety symptoms and anxiety subscales; separation anxiety, social phobia/anxiety, obsessive compulsive, panic and agoraphobia, physical injury fears and generalised anxiety [4, 5]. Preschool Anxiety Scale questionnaires do not assess for panic and agoraphobia symptoms [5].

Incomplete survey responses, from 2 CF (1 Preschool (0–4 years), 1 Parent (5+ years)) and 5 non‐CF controls (1 Preschool (0–4 years), 4 Parent (5+ years)) were excluded from analysis. Completed Spence surveys were scored and total and subscale anxiety scores were calculated and correlated with validated age and gender‐appropriate t‐score. Consistent with previous studies, a *t*‐score cut off of *t* > 60 was selected to represent elevated anxiety symptoms for both total and subscale scores. Health characteristics including modulator use, body mass index (BMI) and lung function represented by forced expiratory volume in 1 s (FEV1%), were collected from clinic records and survey responses to act as markers of health and disease burden.

Statistical analyses were performed using R studio (R v4.3.3). Non‐parametric variables, including demographic variables such as age, gender and BMI were analysed using t‐tests. Cohort scores were analysed using Wilcoxon rank‐sum tests.

Baseline survey results were collected from 41 CF and 43 non‐CF control participants (Table 1). Median (IQR) age at baseline was 6.80 (2.30–12.40) years in the CF cohort and 9.00 (3.80–12.85) years in the non‐CF control cohort. No differences were observed between CF and non‐CF control cohort size (*p* = 0.83), gender (*p* = 0.52), age (*p* = 0.27) or BMI (*p* = 0.28) at baseline.

**Table 1:** CF and Non‐CF Controls Cohort Characteristics.

*Baseline Cohort*				CF	Non‐CF
	***n***			41	43
	***Female (%)***			21 (51%)	18 (42%)
	***Median age at Baseline (IQR)***			6.80 (2.30–12.40)	9.00 (3.80–12.85)
	***Median BMI Z‐score (IQR)***			0.33 (−0.15 to 0.90)	−0.07 (−0.52 to 1.01)
	***Homozygous F508del mutations***			19	NA
	***Modulator Use at Time of Survey***			7 (17.07%)	NA
	***Median % FEV1 (IQR)***			94.00 (85.00–102.00)	
	***Survey Completion***	***Preschool: 0–4 years***	***n* (%)**	16 (39%)	15 (34.9%)
			**Female (%)**	10 (62.5%)	6 (40%)
		***Parent: 5+ years***	***n* (%)**	17 (41.5%)	7 (16.3%)
			**Female (%)**	7 (41%)	3 (43%)
		***Child: 8+ years***	***n* (%)**	8 (19.5%)	21 (48.8%)
			**Female (%)**	4 (50%)	9 (43%)

No significant differences were observed in the total or subscale *t*‐scores in the Preschool (0–4), Parent (5+) and Child (8+) Spence surveys (Supplementary Table 1). In the pooled cohort of participants, 2.43% of CF and 6.98% of non‐CF control participants reported elevated anxiety symptoms (Table 2).

**Table 2:** Baseline Spence Survey Results for All CF and Non‐CF Control participants.

			*t‐score*		
	*Number of elevated t‐scores (%)*		*Mean*		*Wilcoxon test*
	*CF*	*Non‐CF*	*CF*	*Non‐CF*	
*Total Score*	1 (2.43%)	3 (6.98%)	43.10	44.91	W = 743, *p* value = 0.19
*Separation Anxiety*	2 (4.88%)	4 (9.30%)	46.12	46.51	W = 883.5, *p* value = 0.99
*Social Phobia*	1 (2.43%)	4 (9.30%)	43.68	46.33	W = 751.5, *p* value = 0.23
*Obsessive Compulsive*	1 (2.43%)	2 (4.65%)	45.20	45.81	W = 914, *p* value = 0.76
*Panic/agoraphobia* *	1 (4%)	2 (4.65%)	50.12	47.39	W = 421.5, *p* value = 0.20
*Physical Injury Fears*	2 (4.88%)	5 (11.63%)	45.44	47.12	W = 783.5, *p* value = 0.36
*Generalised Anxiety*	3 (7.32%)	4 (9.30%)	45.98	46.19	W = 898, *p* value = 0.88

* Panic/agoraphobia not assessed in Preschool (0–4 years old) survey.

**p< 0.05.

In our study, CF and Non‐CF controls reported low levels of anxiety symptoms. No significant differences in reported anxiety scores were observed between CF and non‐CF control cohorts.

To our knowledge, only one study has previously examined the prevalence of anxiety in CF children aged under twelve [6]. Gundogdu et al. [6] assessed 20 Turkish CF pre‐adolescents (8–11 years old) and 12 CF adolescents (12–16 years old) for anxiety symptoms using the Screen for Child Anxiety and Related Disorders [6]. This group was compared to 20 preadolescent and 13 adolescent controls. In this single‐centre cohort, 46.9% of CF participants experienced anxiety symptoms, compared to 15.2% of controls (*p* = 0.007). CF participants also experienced significantly higher rates of specific phobias, however when stratified by age, this was only observed in pre‐adolescents (*p* = 0.005) [6].

Whilst the prevalence of anxiety symptoms seen in our cohort are lower than that seen by Gundogdu et al, to our knowledge there are no large‐scale cohort studies of similarly aged CF cohorts to compare our findings [6]. Large‐scale international cohorts of 12–18 years surveyed in TIDES likewise reported higher rates of anxiety than that observed in our cohort, of 22% [1]. Variations in screening tools used between these studies further limit comparisons.

We hypothesise that the comparatively low levels of anxiety in our cohort may reflect our cohort's low disease burden, as reflected through their BMI *z*‐scores and FEV1% (Table 1). Scores may also reflect patient's greater access to treatment from multidisciplinary services at our centre, including psychological services.

Although high parent‐child response inter‐reliability has been observed in Spence Anxiety Scale questionnaires, our study is limited by its reliance on subjective recall of participants [4]. In the present study, we did not explicitly correlate anxiety with health and treatment‐related events such as recent pulmonary exacerbation, hospitalisations or medication initiation, nor did we assess for behavioural manifestations of anxiety which may reflect anxiety in younger children. Assessment of potential confounding factors including patient demographics relating to location of residence, socioeconomic status and parent or caregiver anxiety, not analysed here, may have also introduced bias in the recruitment of both CF and non‐CF controls, limiting generalisability. Assessment of these additional variables in future, large‐scale, longitudinal studies will assist with identifying at‐risk subgroups within preadolescent pediatric CF cohorts.

Overall, this single‐centre cohort study found a low prevalence of anxiety symptoms in preadolescents CF patients, which did not significantly differ from rates seen in non‐CF controls. Further studies are required to investigate the need and timing for earlier intervention or screening of CF preadolescents at risk of anxiety.

## Author Contributions

**Jemilla Strode Smith:** writing – original draft, data curation, formal analysis. **Josie van Dorst:** supervision, writing – review and editing, formal analysis. **Michael J. Coffey:** methodology, conceptualization, investigation, writing – review and editing. **Chee Y. Ooi:** funding acquisition, writing – review and editing, supervision, investigation, conceptualization, methodology, project administration.

## Acknowledgments

Open access publishing facilitated by University of New South Wales, as part of the Wiley ‐ University of New South Wales agreement via the Council of Australian University Librarians.

## Ethics Statement

This study is approved by the Sydney Children's Hospital Network Human Research Ethics Committee, Sydney, Australia. Ethics number: HREC/18/SCHN/26.

## Conflicts of Interest

The authors declare no conflicts of interest.

## Supporting information

Supplementary t‐score table.

## Data Availability

The data that support the findings of this study are available from the corresponding author upon reasonable request.
