# Supplementary material for: Anxiety in the Pediatric Cystic Fibrosis Population: Evaluation of a Younger Cohort
Source: Pediatr Pulmonol. 2025 Jun 26;60(6):e71181. doi: 10.1002/ppul.71181 (PMC12202716; doi:10.1002/ppul.71181)
Supplement: Supplementary file 1 — Supplementary t‐score table. [file PPUL-60-0-s001.docx]

**Supplementary Table 1: Anxiety t-score results stratified by survey form completed**

|  | **Preschool** | | | | | **Parent** | | | | | **Child** | | | | | **Pooled Cohort** | | | | | |
| --- | --- | --- | --- | --- | --- | --- | --- | --- | --- | --- | --- | --- | --- | --- | --- | --- | --- | --- | --- | --- | --- |
| ***Sample age range (years)*** | **0-4** | | | | | **5+** | | | | | **8+** | | | | | **0-18** | | | | | |
| *N* | CF = 16, Non-CF = 15 | | | | | CF = 17, Non-CF =7 | | | | | CF = 8, Non-CF =21 | | | | | CF =41, Non-CF =43 | | | | | |
|  | ***Number of elevated t-scores (%)*** | | ***t-score*** | | | ***Number of elevated t-scores (%)*** | | ***t-score*** | | | ***Number of elevated t-scores (%)*** | | ***t-score*** | | | ***Number of elevated t-scores (%)*** | | | ***t-score*** | | |
|  | ***CF*** | ***Non-CF*** | ***Mean*** | | ***Wilcoxon test*** | ***CF*** | ***Non-CF*** | ***Mean*** | | ***Wilcoxon test*** | ***CF*** | ***Non-CF*** | ***Mean*** | | ***Wilcoxon test*** | ***CF*** | | ***Non-CF*** | ***Mean*** | | ***Wilcoxon test*** |
|  |  |  | ***CF*** | ***Non-CF*** |  |  |  | ***CF*** | ***Non-CF*** |  |  |  | ***CF*** | ***Non-CF*** |  |  |  |  | ***CF*** | ***Non-CF*** |  |
| ***Total Score*** | 0 (0%) | 1 (6.67%) | 41.94 | 44.53 | W = 100.5, p-value = 0.41 | 1 (5.88%) | 0 (0%) | 44.24 | 42.57 | W = 54.5, p-value = 0.76 | 0 (0%) | 2 (9.52%) | 43 | 45.95 | W = 69.5, p-value = 0.48 | 1 (2.43%) | 3 (6.98%) | | 43.10 | 44.91 | W = 743, p-value = 0.19 |
| ***Separation Anxiety*** | 1 (6.25%) | 0 (0%) | 47.31 | 47.13 | W = 117, p-value = 0.92 | 1 (5.88%) | 0 (0%) | 46.06 | 45.86 | W = 57.5, p-value = 0.92 | 0 (0%) | 4 (19.05%) | 43.88 | 46.29 | W = 85, p-value = 0.98 | 2 (4.88%) | 4 (9.30%) | | 46.12 | 46.51 | W = 883.5, p-value = 0.99 |
| ***Social Phobia*** | 0 (0%) | 0 (0%) | 41.25 | 44.47 | W = 86.5, p-value = 0.15 | 1 (5.88%) | 0 (0%) | 46.59 | 46.71 | W = 59, p-value = 1 | 0 (0%) | 4 (19.05%) | 42.38 | 47.52 | W = 62.5, p-value = 0.28 | 1 (2.43%) | 4 (9.30%) | | 43.68 | 46.33 | W = 751.5, p-value = 0.23 |
| ***Obsessive Compulsive*** | 0 (0%) | 0 (0%) | 43 | 41.87 | W = 134, p-value = 0.49 | 1 (5.88%) | 0 (0%) | 48.82 | 51.57 | W = 56.5, p-value = 0.86 | 0 (0%) | 2 (9.52%) | 41.88 | 46.71 | W = 56.5, p-value = 0.15 | 1 (2.43%) | 2 (4.65%) | | 45.20 | 45.81 | W = 914, p-value = 0.76 |
| ***Panic/ agoraphobia*** | * | * | * | * | * | 1 (5.88%) | 0 (0%) | 52.47 | 51.29 | W = 69.5, p-value = 0.52 | 0 (0%) | 2 (9.52%) | 45.13 | 46.10 | W = 78.5, p-value = 0.80 | 1 (4%) | 2 (4.65%) | | 50.12 | 47.39 | W = 421.5, p-value = 0.20 |
| ***Physical Injury Fears*** | 0 (0%) | 2 (13.33%) | 42.56 | 44.67 | W = 111, p-value = 0.70 | 1 (5.88%) | 0 (0%) | 47.65 | 44.14 | W = 76.5, p-value = 0.27 | 1 (12.5%) | 3 (14.29%) | 46.5 | 49.86 | W = 61.5, p-value = 0.27 | 2 (4.88%) | 5 (11.63%) | | 45.44 | 47.12 | W = 783.5, p-value = 0.36 |
| ***Generalised Anxiety*** | 0 (0%) | 1 (6.67%) | 41.63 | 44.2 | W = 112, p-value = 0.73 | 2 (11.76%) | 1  (14.29%) | 49.35 | 48.57 | W = 59.5, p-value = 1 | 1 (12.5%) | 2 (9.52%) | 47.5 | 46.81 | W = 100.5, p-value = 0.42 | 3 (7.32%) | 4 (9.30%) | | 45.98 | 46.19 | W = 898, p-value = 0.88 |
